# Four new species of splanchnotrophid copepods (Poecilostomatoida) parasitic on doridacean nudibranchs (Gastropoda, Opistobranchia) from Japan, with proposition of one new genus

**DOI:** 10.3897/zookeys.247.3698

**Published:** 2012-11-30

**Authors:** Daisuke Uyeno, Kazuya Nagasawa

**Affiliations:** 1 Faculty of Science, University of the Ryukyus, 1 Senbaru, Nishihara, Okinawa 903-0213, Japan; 2 Graduate School of Biosphere Science, Hiroshima University, 1-4-4 Kagamiyama, Higashi-Hiroshima, Hiroshima 739-8528, Japan

**Keywords:** ParasiticCopepoda, Splanchnotrophidae, *Ceratosomicola*, *Splanchnotrophus*, *Majimun*, new genus, sea slugs

## Abstract

Four new species of splanchnotrophid copepods are described based on specimens collected from 5 species of doridacean nudibranchs from coastal waters of Japan. They belong to 3 genera, one of which, *Majimun* gen. n., is new. The parasites and their hosts are as follows: *Ceratosomicola japonica* **sp. n.** ex *Hypselodoris festiva* (A. Adams); *Splanchnotrophus helianthus* **sp. n.** ex *Thecacera pennigera* (Montagu); *Splanchnotrophus imagawai* **sp. n.** ex *Trapania miltabrancha* Gosliner & Fahey; and *Majimun shirakawai* **gen. et sp. n.** ex *Roboastra luteolineata* (Baba) and *Roboastra gracilis* (Bergh). *Ceratosomicola japonica* **sp. n.** is the fifth species of *Ceratosomicola* and is characterized by the shape and armature of the prosome in females. Both *Splanchnotrophus helianthus* **sp. n.** and *Splanchnotrophus imagawai* **sp. n.** are differentiated from 4 known congeners by the absence of posterolateral processes or lobes on the prosome in females, and the females of these 2 new species are separated from each other by the shape and armature of the genito-abdomen, the mandible, and the swimming legs. *Majimun* **gen. n.** is distinguished from other splanchnotrophid genera by the segmentation of the antennule as well as the combination of the following characters in females: 2 postgenital somites and the shape of the antenna, the mandible and the swimming legs.

## Introduction

The Splanchnotrophidae is a bizarre copepod family of Poecilostomatoida. Its members parasitize marine opisthobranch gastropods (Huys 2001). It is often difficult to detect their presence because almost all parts of the parasite’s body are usually embedded inside the host, and only the distal part of the urosome and the egg sacs are exposed and visible externally. Since the mid-19th century, splanchnotrophids have been reported or described by many malacologists in the course of studies on opisthobranch gastropods (see Huys 2001). The taxonomic studies have often been inadequate at the generic level because the original descriptions include errors or have omissions. In his revision of Splanchnotrophidae, Huys (2001) clarified the validity of 3 genera, *Splanchnotrophus* Hancock & Norman, 1863, *Ismaila* Bergh, 1868, and *Lomanoticola* Scott T. & A., 1895, and established 2 new genera, *Arthurius* Huys, 2001 and *Ceratosomicola* Huys, 2001. Since then, 1 species of *Arthurius*, 8 of *Ismaila*, and 3 of *Ceratosomicola* have been described, and a total of 23 species belonging to 5 genera are recognized in this family at present (Haumayr and Schrödl 2003; Salmen et al. 2008a, b). In this study, 4 new species of splanchnotrophid copepods collected from Japanese waters are described. Based on one new species, a new genus *Majimun* gen. n. is herein established.

## Material and methods

Doridacean nudiblanchs were collected by SCUBA diving in the Seto Inland Sea off Hiroshima, central Japan and in both the North Pacific Ocean and the East China Sea off Okinawa-jima Island, the Central Ryukyu Islands, southern Japan, from April 2008 to December 2010. Collection data including the numbers of copepods found on the nudibranchs examined are shown in Table 1. Copepods were carefully removed from the body cavities of the hosts via dissections and preserved in 80% ethanol. Specimens were soaked in lactophenol for 2 days before dissection. The appendages of the copepods were then dissected and observed using the method of Humes and Gooding (1964). The drawings were made with the aid of a drawing tube. Morphological terminology follows Huys and Boxshall (1991) and Huys (2001). Measurements in millimeters are shown as ranges in parentheses with means and standard deviations. Type specimens are deposited in the crustacean collection of the National Museum of Nature and Science, Tsukuba (NSMT) and the Ryukyu University Museum, Fujukan (RUMF), Okinawa. The scientific names of nudibranchs follow those listed by Debelius and Kuiter (2007), Gosliner et al. (2008), and Gosliner and Fahey (2008).

**Table 1.** Collection data of the nudibranchs infected by splanchnotrophid copepods examined in present study.

**Host nudibranch**	**Number of hosts examined**	**Locality**	**Date**	**Copepod**	**Number of copepod specimens**
*Hypselodoris festiva*	1	Off Irukabana, Nohmi-jima Island, Hiroshima, Seto Inland Sea (34°13'49"N, 132°23'7"E), 5 m	11 Dec. 2010	*Ceratosomicola japonica*	2♀, 4 ♂
	2	Off Irukabana, Nohmi-jima Island, Hiroshima, Seto Inland Sea (34°13'49"N, 132°23'7"E), 3 m	12 Dec. 2010	*Ceratosomicola japonica*	1♀, 2♂
*Thecacera pennigera*	1	Off Izaki, Yashiro-jima Island, Yamaguchi, Seto Inland Sea (33°51'49"N, 132°19'29"E), unknown depth	27 Apr. 2008	*Splanchnotrophus helianthus*	1♀,1♂
	2	Off Matoba Beach, Takehara, Hiroshima, Seto Inland Sea (34°19'29"N, 132°55'21"E), 15 m	15 Jan. 2009	*Splanchnotrophus helianthus*	1♀
	3	Off Matoba Beach, Takehara, Hiroshima, Seto Inland Sea (34°19'29"N, 132°55'21"E), 15 m	17 Feb. 2009	*Splanchnotrophus helianthus*	1♀, 10 ♂
	4	Off Matoba Beach, Takehara, Hiroshima, Seto Inland Sea (34°19'29"N, 132°55'21"E), 15 m	17 Feb. 2009	*Splanchnotrophus helianthus*	1♀
*Trapania miltabrancha*	1	Off Red Beach, Kin, Okinawa-jima Island, North Pacific Ocean (26°26'41"N, 127°54'39"E), 15 m	29 May 2008	*Splanchnotrophus imagawai*	1♀
	2	Off Red Beach, Kin, Okinawa-jima Island, North Pacific Ocean (26°26'41"N, 127°54'39"E), 15 m	23 Apr. 2009	*Splanchnotrophus imagawai*	1♀
*Roboastra luteolineata*	1	Off Miyagi Beach, Chatan, Okinawa-jima Island, East China Sea (26°19'44"N, 127°44'35"E), unknown depth	14 Oct. 2009	*Majimun shirakawai*	2♀, 1♂
	2	Off Miyagi Beach, Chatan, Okinawa-jima Island, East China Sea (26°19'44"N, 127°44'35"E), 6 m	14 Jun. 2010	*Majimun shirakawai*	1♀, 1♂
*Roboastra gracilis*	1	Off Cape Maeda, Onna, Okinawa-jima Island, East China Sea (26°26'41"N, 127°46'20"E), 5 m	Jun. 2010	*Majimun shirakawai*	1♀, 1 ♂

## Results

### Family Splanchnotrophidae Norman & Scott, 1906

*Ceratosomicola* Huys, 2001

#### Ceratosomicola japonica sp. n.

New Japanese name: umiushi-yadori for the family, the genus, and the species

urn:lsid:zoobank.org:act:C4997364-5CBC-4B04-9C1E-8DD13111C5C9

http://species-id.net/wiki/Ceratosomicola_japonica

**Figure 1.**
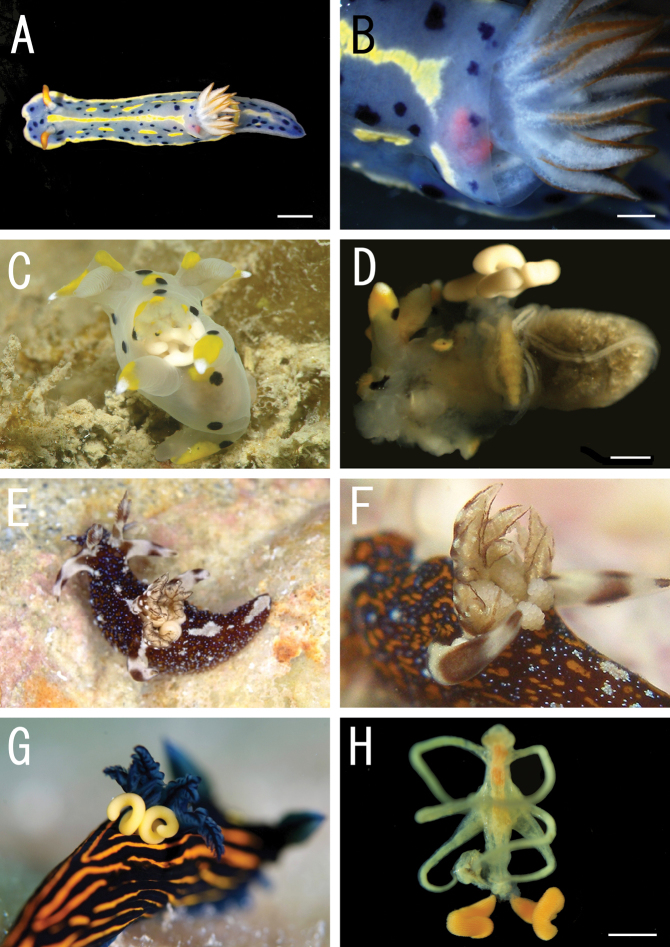
Live coloration of the host nudibranchs and the splanchnotrophids. **A** *Hypselodoris festiva* infected by an ovigerous specimenof *Certosomicola japonica* sp. n. **B** an egg sac of *Ceratosomicola japonica* sp. n. and the gill circle of *Hypselodoris festiva* with the mantle malformed into an elongate tube **C** *Thecacera pennigera* infected by an ovigerous specimen of *Splanchnotrophus helianthus* sp. n. **D** *Trapania pennigera* with the mantle removed to show a female specimen of *Splanchnotrophus helianthus* on the visceral sac **E** *Trapania miltabrancha* infected by an ovigerous specimen of *Splanchnotrophus imagawai* sp. n (photo by K. Imagawa) **F** gill circle of *Trapania miltabrancha* with egg sacs of *Splanchnotrophus imagawai* sp. n. (photo by K. Imagawa) **G** *Roboastra luteolineata* infected by an ovigerous specimen of *Majimun shirakawai* gen. et sp. n. (photo by N. Shirakawa) **H** female *Majimun shirakawai* gen. et sp. n. with dwarf male attached to the posterior part of the body. Scale bars = 5 mm in **A**; 1 mm in **B, D, H**.

Figures 1A, B 2 [Fig F3] 4

##### Type material.

Holotype: female, ex body cavity of *Hypselodoris festiva* (A. Adams) (Nudibranchia: Chromodorididae), off Irukabana, Nohmi-jima Island, Hiroshima, Seto Inland Sea, Japan (34°13'49"N, 132°23'7"E), 5 m depth, 11 December 2010 (NSMT–Cr 22240). Allotype: male (NSMT–Cr 22241), collection data same as that of holotype. Paratypes: 1 female and 3 males (NSMT–Cr 22242), collection data same as that of holotype; 1 female and 2 males ex body cavity of *Hypselodoris festiva*, off Irukabana, Nohmi-jima Island, Hiroshima, Seto Inland Sea, Japan (34°13'49"N, 132°23'7"E), 3 m depth, 12 December 2010 (NSMT–Cr 22243).

##### Type locality.

Off Irukabana, Nohmi-jima Island, Hiroshima, Seto Inland Sea, Japan (34°13'49"N, 132°23'7"E).

##### Description of holotype female.

**Figure 2.**
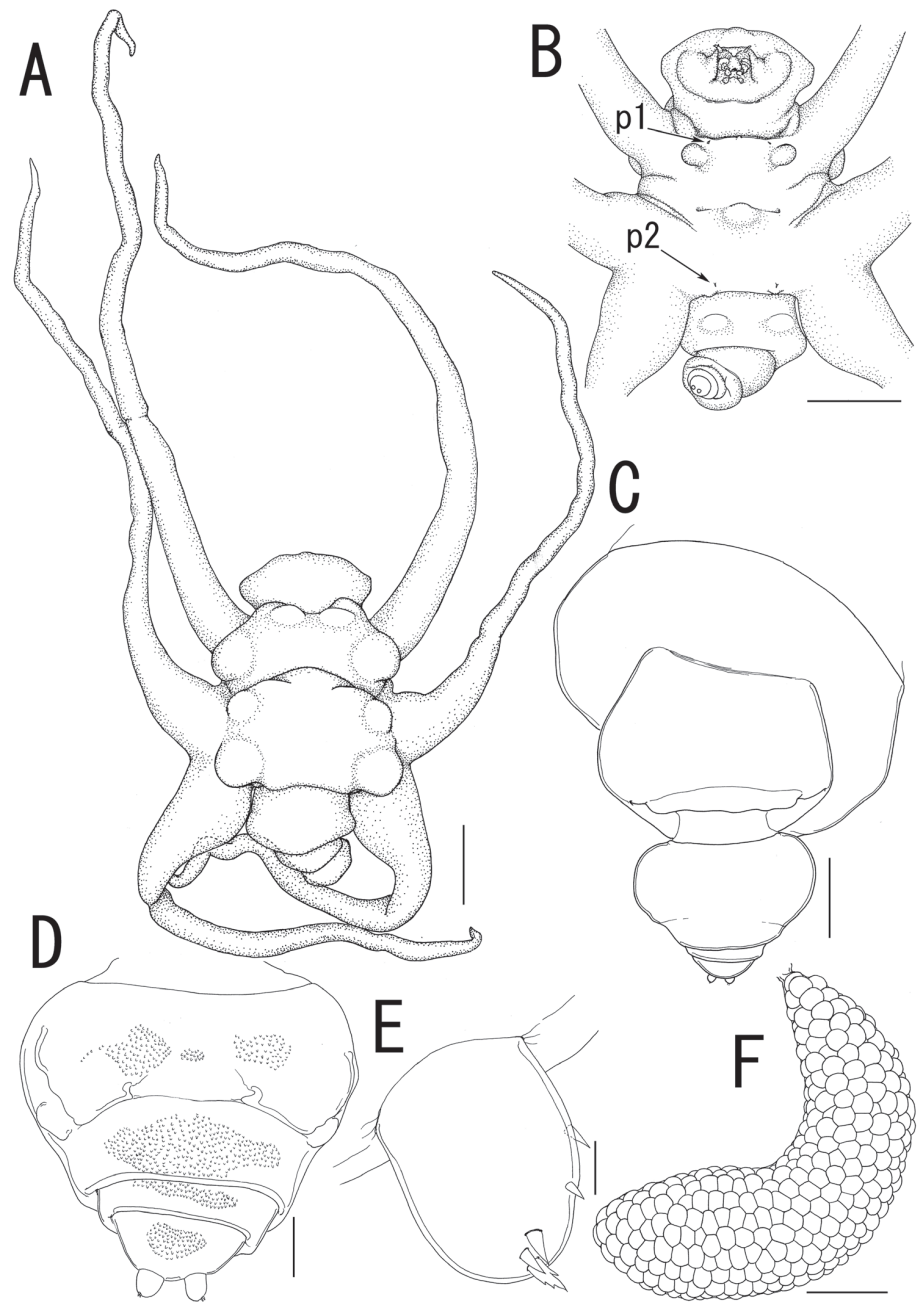
*Ceratosomicola japonica* sp. n., female, holotype NSMT–Cr 22240 (**A–C**), female, paratype NSMT–Cr 22243 (**D–F**). **A** habitus dorsal **B** habitus, ventral, p1 = leg 1, p2 = leg 2 **C** posterior portion of body, ventral **D** urosome, ventral **E** caudal ramus, ventral **F** egg sac. Scale bars = 1 mm in **A, B**; 300 μm in **C**; 100 μm in **D**; 10 μm in **E**; 500 μm in **F**.

**Figure 3. F3:**
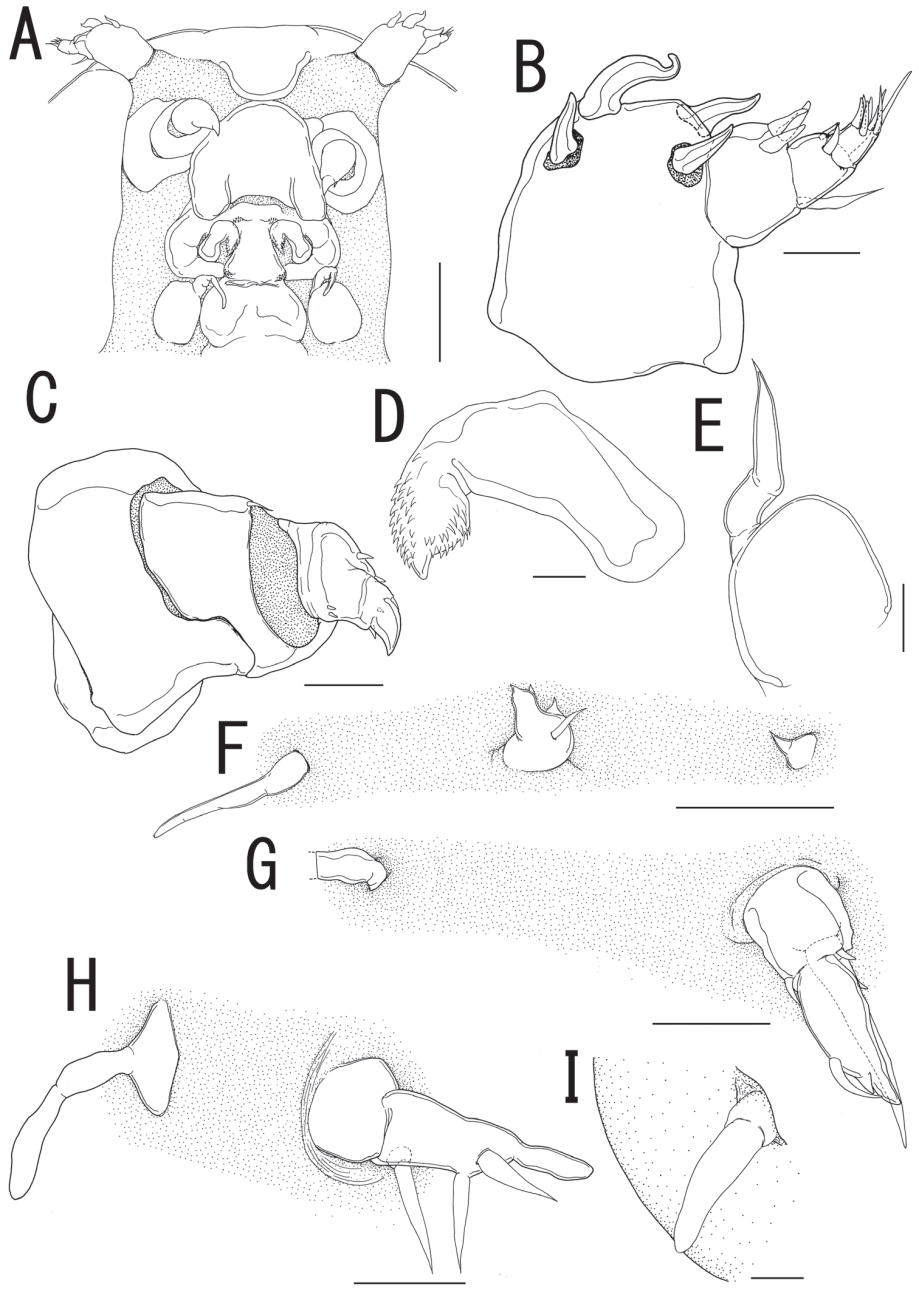
*Ceratosomicola japonica* sp. n., female, holotype NSMT–Cr 22240 (**A–G**), female, paratype NSMT–Cr 22243 (**H**), female, paratype NSMT–Cr 22242 (**I**). **A** cephalosome, anterior portion. ventral **B** antennule, anterior **C** antenna **D** mandible **E** maxilla **F** leg 1 **G** leg 2 **H**, leg 2 (drawn from a paratype, NSMT–Cr 22243) **I** leg 3. Scale bars = 100 μm in **A**; 20 μm in **B**; 30 μm in **C, G, H**; 10 μm in **D, I**; 50 μm in **E, F**.

Body length from rostrum to posterior margin of anal somite: 4.27. Body (Figure 2A) composed of large prosome with 3 pairs of ventrolateral processes and small 3-segmented urosome. Prosome indistinctly 3-segmented, composed of anterior region, cephalosome, middle region comprising first to second pedigerous somites, and posterior region as third and fourth pedigerous somites. Cephalosome (Figures 2A, B, 3A) ellipsoid bearing rostrum with round margin, wider than long, bearing single apical lobe and 1 paired lateral lobes. Middle region large, bearing two transverse dorsal bulges and 5 ventral protrusions; anterodorsal bulge ornamented by 2 paired anterior and 1 paired lateral protrusions; posterior dorsal bulge carrying 2 pairs of lateral protrusions. Posterior region (Figure 2A–C) bearing two ventral protrusions on third pedigerous somite and constriction at border between third and fourth pedigerous somites. Ventrolateral processes (Figure 2A) long and slender, distinctly longer than body. Urosome (Figure 2D) onion-like shaped, comprising genital double somite and two free postgenital somites ornamented with pattern of small scales on ventral surface. Genital double somite bearing paired ventral genital apertures. Caudal rami (Figure 2E) globular bearing two and three spiniform elements on outer margin and tip, respectively; one element on tip serrated.

Antennule (Figure 3A, B) 4-segmented; proximal segment rectangular bearing 4 spines on anterior margin; second segment with 3 anterior spiniform and 1 posterior setiform elements; third segment bearing 2 anterior and 1 posterior elements; terminal segment bearing 6 spiniform and 1 setiform elements. Antenna (Figure 3A, C) 3-segmented, conical with large sclerite at base, comprising coxobasis and 2-segmented endopod; coxobasis unarmed; proximal endopodal segment bearing 1 seta; terminal endopodal segment claw-like bearing 7 small elements. Labrum (Figure 3A) bilobate, unarmed. Labium (Figure 3A) bearing two paired spinulose lobes. Mandible (Figure 3A, D) represented by single recurved blade covered with numerous spinules along both anterior and posterior margin. Maxillule absent. Maxilla (Figure 3A, E) weakly sclerotized globular tapering into lanceolate tip. Maxilliped absent.

Swimming legs rudimentary; protopod largely incorporated into ventral wall of prosome. Leg 1 (Figures 2B, 3F) represented by outer basal seta, small exopodal lobe with seta and conical process along outer margin and 2 processes on tip, and spiniform endopodal element. Leg 2 (Figures 2B, 3G) bearing basal seta, elongate exopod indistinctly 2-segmented, tapering into apical process with 4 elements and single process, and endopodal lobe elongate, unarmed with intermedial constriction. Leg 3 on holotype indistinct.

Egg sacs (Figure 2F) curved, semicircle; color in life crimson.

*Variation of female morphology*. The morphology of female paratypes is as in the holotype, except leg 2 shows variability. Leg 3 is distinctly visible on the paratype females. Paratype female (NSMT-Cr 22243) has the exopod of leg 2 (Figure 3H) tapering into apical process with constriction and 2 elements. Paratype female (NSMT-Cr 22242) possesses a vestigial leg 3 (Figure 3I), represented by a blunt element on a protrusion. The specimens from type series (n = 3) range from 3.11–4.27 (3.76 ± 0.59) in body length (BL).

##### Description of allotype male.

**Figure 4.**
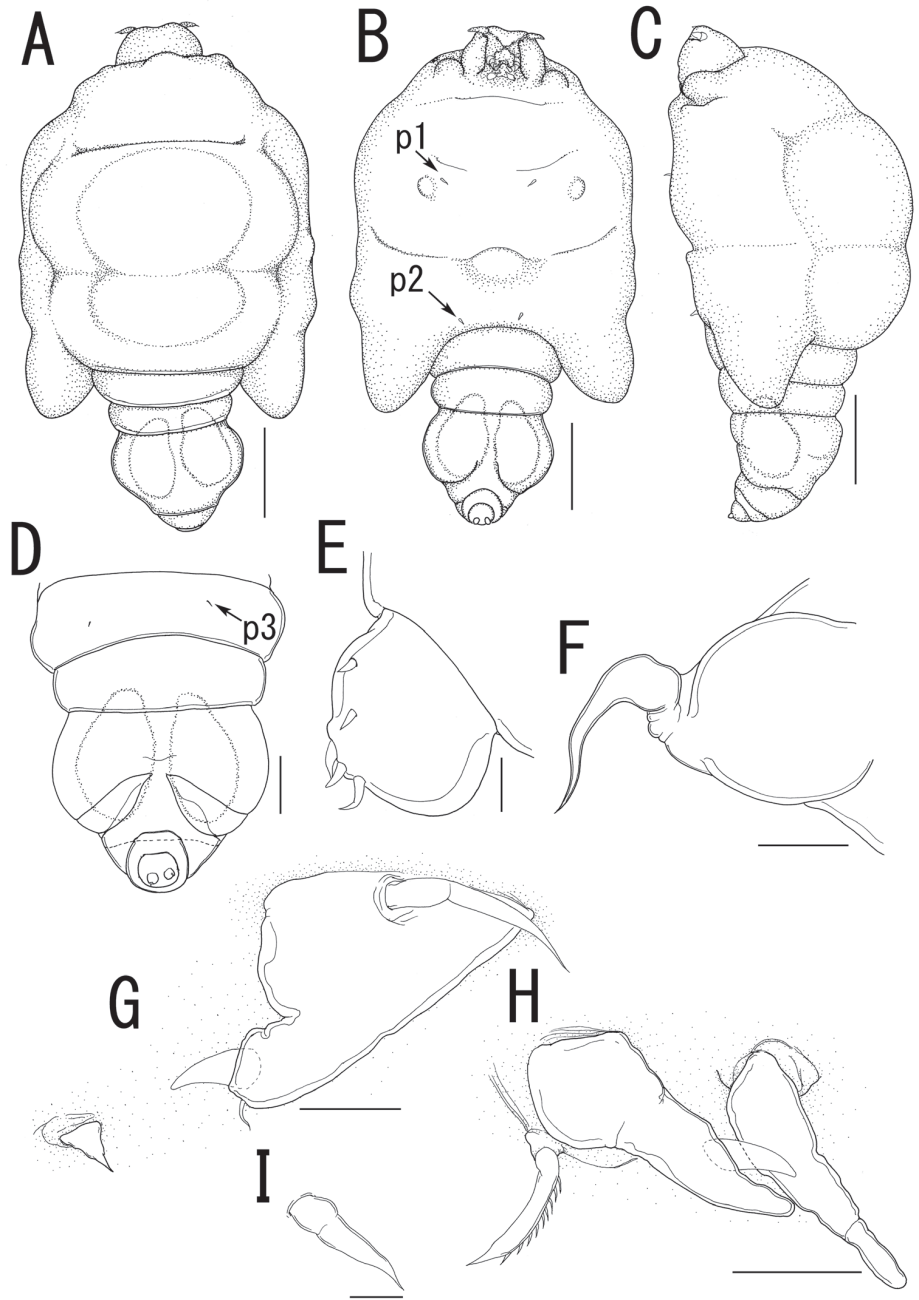
*Ceratosomicola japonica* sp. n., male, allotype NSMT–Cr 22241. **A** habitus, dorsal **B** habitus, ventral, p1 = leg 1, p2 = leg 2 **C** habitus, lateral **D** free thoracic somites and abdomen, ventral, p3 = leg 3 **E** caudal ramus, dorsal **F** maxilla **G** leg 1 **H** leg 2 **I** leg 3. Scale bars = 500 μm in **A, B, C**; 200 μm in **D**; 10 μm in **E, I**; 20 μm in **F, G**; 30 μm in **H**.

Sexual dimorphism present in body form, and swimming legs. Body (Figure 4A–C) 2.81 long, composed of cephalothorax and 5 cylindrical somites. Cephalothorax large, bulbous, incorporating first and second pedigerous somites, bearing transverse constriction and paired lateral and single dorsal protrusions posterior to mouthparts, paired posterolateral outgrowth, and paired and single ventral protrusions. Genital somite (Figure 4D) incompletely segmented, bearing transverse dorsal folding and paired apertures; opercula unarmed. Caudal rami (Figure 4E) globular, 2 and 3 elements along outer margin and on tip, respectively. No marked sexual dimorphism in antennule, antenna, and mouthparts. Tip of maxilla (Figure 4F) slightly sharper than that of female.

Leg 1 (Figure 4B, G) represented by outer basal seta, lobate exdopod with 2 elements, and spiniform endopodal element. Leg 2 (Figure 4B, H) represented by outer basal, serrated seta, elongate exopodal lobe with single element, and elongate endopodal lobe with single blunt element on tip. Leg 3 (Figure 4D, I) represented by spiniform element.

*Variation of male morphology*. The morphology of male paratypes is as in the allotype. The specimens from type series (n = 6) range from 2.16-2.81 (2.42 ± 0.43) in BL.

##### Site.

Female and male specimens were found in the body cavity of the host nudibranchs. Only the posterior tip of the urosome and the egg sacs were exposed from the host’s gill circle (Figure 1A, B). The mantle around the gill circle of the infected nudibranch was malformed into elongate tubes which obscured the host’s gills and the egg sacs of the copepod (Figure 1B).

##### Etymology.

The specific name of the new species “*japonica*” refers to Japan, where it was collected. *Hypselodoris festiva*, the type host of this new species, is widely distributed around the Japanese archipelago and is one of the common nudibranchs of Japan. *Ceratosomicola japonica* sp. n. is the first species of parasitic copepods to have been described from Japan (Fujita 1895).

##### Remarks.

*Ceratosomicola sacculata* (O’Donoghue, 1924) was originally described as *Splanchnotrophus sacculatus*. Huys (2001) redescribed this species based on a female and established a new genus Ceratosomicola. Three species, *Ceratosomicola coia* Salmen, Wilson & Schrödl, 2008; *Ceratosomicola delicata* Salmen, Wilson & Schrödl, 2008; *Ceratosomicola mammilata* Salmen, Wilson & Schrödl, 2008, were subsequently described based on specimens of both sexes, and this genus is now composed of four species (Salmen et al. 2008b). The new species clearly differs from *Ceratosomicola coia* and *Ceratosomicola delicata* in having 7 ventral protrusions on the prosome of the female (vs. without ventral protorusions on *Ceratosomicola coia* and *Ceratosomicola delicata*; Salmen et al. 2008b). Although the female of *Ceratosomicola mammilata* shares 7 protrusions, this species can be differentiated from the new species by having 2 pairs of lateral lobes on the anterior region of the prosome and a posterior pair of ventral protrusions located anterior to the base of third ventrolateral processes, i.e. on the second pedigerous somite (vs. 1 pair of lateral lobes on the anterior region of the prosome and a posterior pair of ventral lobes located posterior to the base of third ventrolateral processes, i.e. on the third pedigerous somite). In Huys’ (2001) redescription of *Ceratosomicola sacculata*, the ventral protrusions on the prosome was not described, while in the original description, O’Donoghue (1924) referred to the presence of at least 2 paired ventral lobes on the prosome. However, *Ceratosomicola sacculata* is distinguishable from the new species by the following characters in females: the anterior region of the prosome is trilobate (vs. ellipsoidal and bearing a pair of lateral lobes in the new species) and the middle region of the prosome bears 3 transverse dorsal bulges (vs. 2 transverse dorsal bulges in the new species).

In the course of dissection to describe *Hypselodoris festiva* (as *Chromodoris marenzalleri*) (Nudibranchia: Chromodorididae) from the western North Pacific Ocean off Misaki, Kanagawa Japan (Fujita 1893), one female specimen of splanchnotrophid was discovered by Fujita (1895) from the body cavity of the host. Although Fujita (1895) recognized some differences between this copepod and other splanchnotrophids, he did not describe it as a new species nor deposit it in any museum because of the incomplete specimen. The species was subsequently recognized as a member of *Ceratosomicola* by Huys (2001). *Ceratosomicola japonica* sp. n. was collected from the same host species (*Hypselodoris festiva*) in the Seto Inland Sea off Nohmi-jima Island, Hiroshima, Japan and shares important characters as follows: the anterior region of the prosome is wider than long, bearing a pair of lateral lobes (Fujita 1895, figure 2) and middle region of the prosome bears a paired anterior and single posterior ventral protrusions with the latter being larger than the others (Fujita 1895, figure 1). Fujita (1895, figure 2) also described the middle region of the prosome as bearing a cross-shaped concavity. This corresponds to the transverse dorsal bulges with 4 bulbous protrusions on each corner of *Ceratosomicola japonica* sp. n. Therefore, the Fujita’s splanchnotrophid is apparently conspecific with *Ceratosomicola japonica* sp. n.

### *Splanchnotrophus* Hancock & Norman, 1863

#### Splanchnotrophus helianthus sp. n.

New Japanese name: himawari-umiushi-yadori for both the genus and the species

urn:lsid:zoobank.org:act:744FEC5A-C377-442C-94CC-3F56132053E0

http://species-id.net/wiki/Splanchnotrophus_helianthus

Figures 1C, D 5 [Fig F6] 7

##### Type material.

Holotype: female, ex body cavity of *Thecacera pennigera* (Montagu) (Nudibranchia: Polyceridae), off Matoba Beach, Takehara, Hiroshima, Seto Inland Sea, Japan (34°19'29"N, 132°55'21"E), 15 m depth, 17 February 2009 (NSMT–Cr 22244). Allotype: male (NSMT–Cr 22245), collection data same as that of holotype. Paratypes: 1 female and 9 males (NSMT–Cr 22246), collection data same as that of holotype; 1 female ex body cavity of *Thecacera pennigera*, off Matoba Beach, Takehara, Hiroshima, Seto Inland Sea, Japan (34°19'29"N, 132°55'21"E), 15 m depth, 15 January 2009 (NSMT–Cr 22247); 1 female and 1 male ex body cavity of *Thecacera pennigera*, off Izaki, Yashiro-jima Island, Yamaguchi, Seto Inland Sea, Japan (33°51'49"N, 132°19'29"E), unknown water depth, 27 April 2008 (NSMT–Cr 22248).

##### Type locality.

Off Matoba Beach, Takehara, Hiroshima, Seto Inland Sea, Japan (34°19'29"N, 132°55'21"E).

##### Description of holotype female.

**Figure 5.**
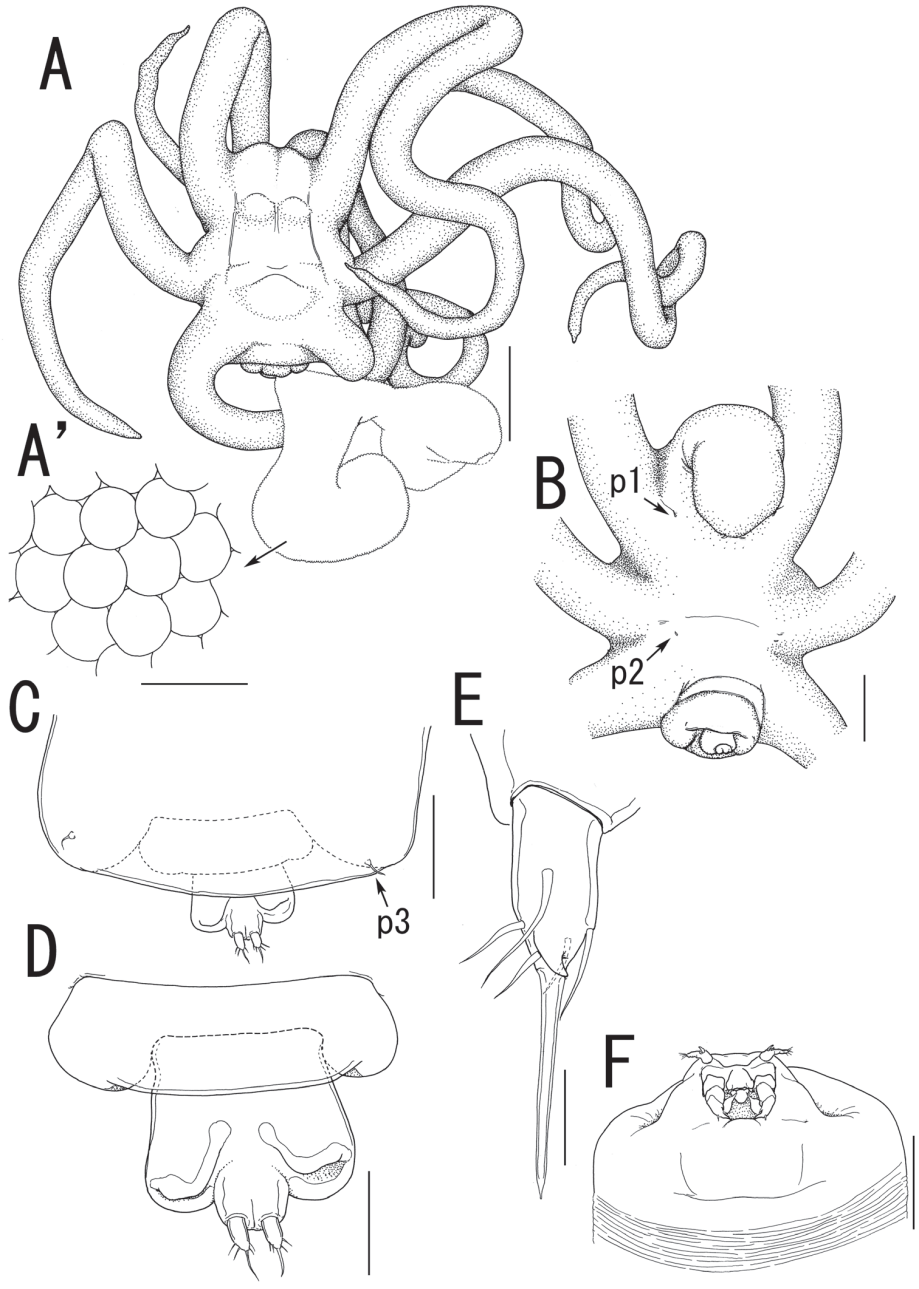
*Splanchnotrophus helianthus* sp. n., female, holotype NSMT–Cr 22244. **A** habitus, dorsal **A’** enlarged view of egg sac **B** habitus, ventral, p1 = leg 1, p2 = leg 2 **C** posterior portion of body, ventral, p3 = leg 3 **D** fourth pedigerous somite and genito-abdomen, ventral **E** caudal ramus, dorsal **F** cephalosome, ventral. Scale bars = 1 mm in **A**; 100 μm in **A’**; 500 μm in **B**; 200 μm in **C**, **F**; 100 μm in **D**; 20 μm in **E**.

Body (Figure 5A) 3.44 long, composed of elongate, slender prosome with 3 pairs of long lateral processes and small 2-segmented urosome. Prosome (Figure 5A, B) composed of anterior region, cephalosome, middle region comprising first to second pedigerous somites, and posterior region as third pedigerous somite. Cephalosome (Figure 5B, F) elongate, bent ventrally, with projecting rostral area. Middle region (Figure 5A, B) large, constricted posterior to base of anterior lateral processes with paired and single dorsal protrusions. Posterior region (Figure 5A–C) broad, without armature. Lateral processes (Figure 5A) long and slender, distinctly longer than body length. Urosome (Figure 5C, D) small; genito-abdomen narrower posteriorly with paired posterolateral lobes; unarmed opercula and genital aperture located on ventral. Caudal rami (Figure 5E) small, about twice as long as wide, bearing 6 setae and 1 dorsal spiniform process; apical seta long, styliform.

**Figure 6. F6:**
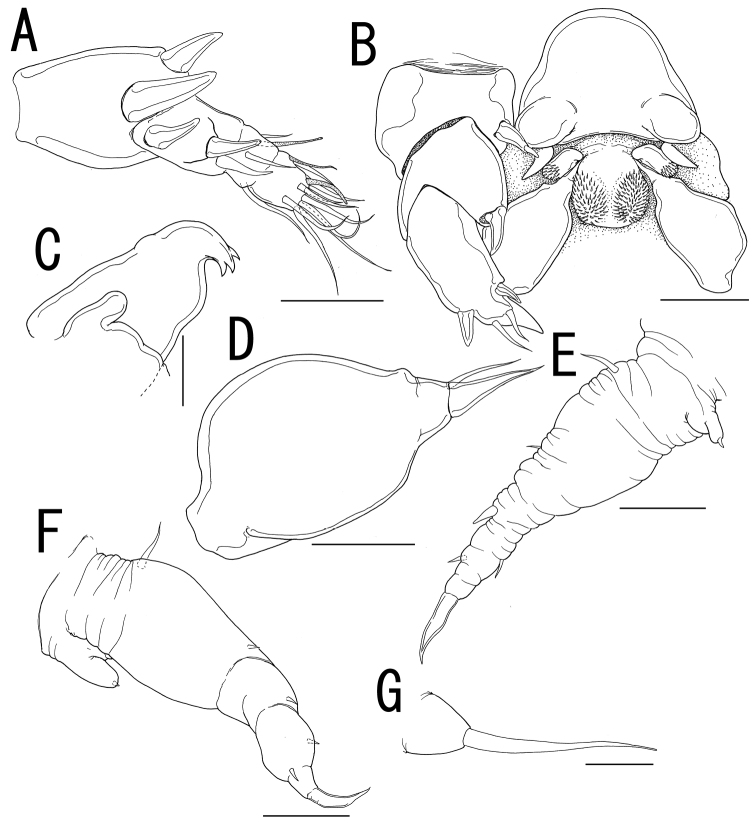
*Splanchnotrophus helianthus* sp. n., female, holotype NSMT–Cr 22244. **A** antennule, anterior **B** oral area **C** mandible, posterior **D** maxilla **E** leg 1 **F** leg 2 **G** leg 3. Scale bars = 20 μm in **A, E, F**; 30 μm in **B**; 10 μm in **C, D, G**.

Antennule (Figures 5F, 6A) 2-segmented; terminal segment bearing 2 constrictions making it appearing as original segmentation; proximal segment bearing 2 blunt spines; terminal segment bearing 2 blunt spines and 1 seta in proximal part, 3 setae and 1 aesthetasc in middle part, and 9 setae and 2 aesthetascs in distal part. Antenna (Figures 5F, 6B) 3-segmented; coxo-basis broad, bearing 1 inner spine with spiniform tip; proximal segment of endopod bearing 1 inner spine; terminal segment of endopod tapering into strong apical claw, with 2 spines and 2 setal elements. Labrum (Figure 6B) bilobate, bearing flat surface. Mandible (Figure 6C) spatulate, tapering into single curved blade with 2 dentiform processes giving trifid appearance. Labium (Figure 6B) developed with paired spinulose patches. Maxillule not observed. Paragnath (Figure 6B) developed, represented by pinnate lobe. Maxilla (Figure 6B, D) 2-segmented; syncoxa unarmed; allobasis tapering into spiniform element, with seta. Maxilliped absent.

Leg 1(Figures 5B, 6E) unsegmented, weakly sclerotized and drawn out into elongate exopod and small endopod; protopod bearing outer basal seta; exopod drawn out into spiniform lobe bearing multiple constrictions, wrinkly surface, 3 outer and 1 inner setal elements; endopod a small lobe tipped with seta. Leg 2 (Figures 5B, 6F) unsegmented, weakly sclerotized; protopod drawn out into long exopod and small, cylindrical endopod; protopod bearing outer basal seta; exopod tapering into a pointed process with three outer and 1 inner small element; endopodal lobe bearing small apical seta. Leg 3 (Figures 5C, 6G) represented by conical process with apical seta, located near posterolateral corner on ventral side of prosome.

Egg sacs (Figure 5A) bilobate, bearing curved side and swollen side; color in life cream (Figure 1C, D).

*Variation of female morphology*. The morphology of female paratypes is as in the holotype. The specimens from type series (n = 3) range from 2.81–4.47 (3.57 ± 0.83) BL.

##### Description of allotype male.

**Figure 7.**
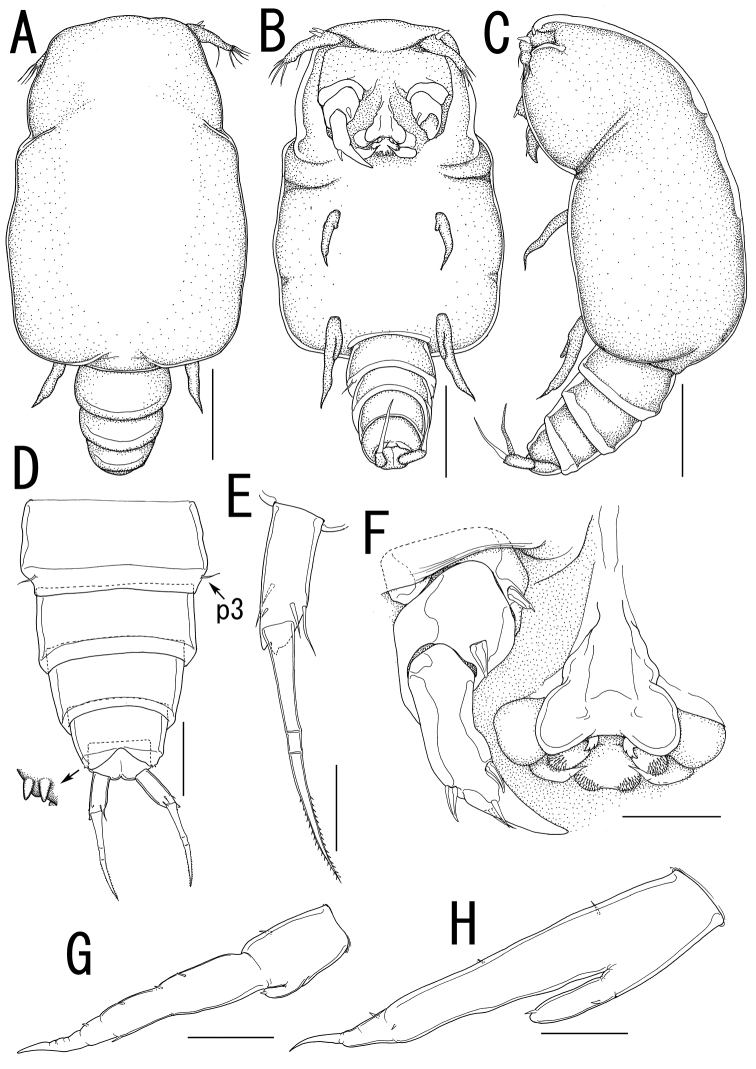
*Splanchnotrophus helianthus* sp. n., male, allotype NSMT–Cr 22245. **A** habitus, dorsal **B** habitus, ventral **C** habitus, lateral **D** free thoracic somites and abdomen, ventral **E** caudal ramus, ventral **F** oral area **G** leg 1 **H** leg 2. Scale bars = 100 μm in **A, B, C**; 50 μm in **D**; 20 μm in **E**.

Sexual dimorphism prominent in body form. Body (Figure 7A–C) cyclopiform, 0.63 long, composed of cephalothorax and 5 cylindrical somites. Cephalothorax (Figure 7A–C) large, incorporating first and second pedigerous somites, with constriction posterior to mouthparts. Urosome 3-segmented (Figure 7D); genital somite scarcely discernible in dorsal view, bearing paired apertures; opercula carrying 2 processes along posterior margin. Anal somite (Figure 7D) nearly completely withdrawn into genital somite. Caudal rami (Figure 7E) cylindrical, about three times as long as wide, bearing 5 setae, styliform terminal seta bipinnate toward tip, and 2 dorsal spiniform spines.

No marked sexual dimorphism in antennule, antenna, and mouth parts, except location of antenna. The base of antenna located anterior to labrum (Figure 7F).

Leg 1 (Figure 7G) biramous; protopod narrower than that of female, with minute basal outer seta; exopodal lobe elongate, tapering into pointed process, carrying 4 outer and 1 inner elements; endopodal lobe small, tipped with minute apical element. Leg 2 (Figure 7H) longer than leg 1; protopod bearing minute basal outer seta; exopodal lobe elongate, tapering into pointed process, bearing 3 outer and 1 inner elements; endopodal lobe tipped with minute element, bearing 1 small outer element. Leg 3 (Figure 7D) represented by single seta. Legs 4 and 5 absent.

*Variation of male morphology*. The morphology of male paratypes is as in the allotype. The specimens from type series (n = 11) range from 0.32–0.63 (0.53 ± 0.12) in BL.

##### Site.

Both female and male specimens were found in the body cavity of host nudibranchs. The females grasped the host’s visceral sac by the lateral processes on the prosome (Figure 1D). Only the posterior tip of the urosome and the egg sacs were exposed from the host’s gill circle (Figure 1C).

##### Etymology.

The specific name “*helianthus*” is from the Latin meaning sunflower. The live body color of this new species is yellowish, and the egg sacs attached on the host nudibranch look like flowers.

##### Remarks.

Four species of *Splanchnotrophus* are currently recognized as valid (Huys 2001). *Splanchnotrophus helianthus* sp. n. differs from *Splanchnotrophus angulatus* Hecht, 1893, *Splanchnotrophus dellachiajei* Delamare Deboutteville, 1950, *Splanchnotrophus gracilis* Hancock & Norman, 1863 in the absence of paired posterolateral processes on the prosome and the genito-abdomen bearing lateral lobes in females (vs. the presence of posterolateral processes on the prosome and the genito-abdomen without paired lateral lobes, Hancock and Norman 1863; Delamare Deboutteville 1950; Huys 2001). Huys (2001) claimed that, in *Splanchnotrophus angulatus*, the shape of the female’s genito-abdomen is constant, irrespective of prosome valiability, and the size and shape of the posterolateral lobe of the prosome certainly shows variability. Nevertheless, this species always possesses the posterolateral lobe, which is regarded as a useful identification character. The original description of *Splanchnotrophus willemi* by Canu (1891) has no illustration and includes only a minimum amount of information. However, the presence of pleural wings on the third pedigerous somite in *Splanchnotrophus willemi* is not shared with the new species (Canu 1891).

#### Splanchnotrophus imagawai sp. n.

New Japanese name: uzu-himawari-umiushi-yadori

urn:lsid:zoobank.org:act:5DD5D5DA-2D24-4A69-A64D-6C3A5004920D

http://species-id.net/wiki/Splanchnotrophus_imagawai

Figures 1E, F 8 9

##### Type material.

Holotype: female, ex body cavity of *Trapania miltabrancha* Gosliner & Fahey (Nudibranchia: Goniodorididae), off Red Beach, Kin, Okinawa-jima Island, North Pacific Ocean, Japan (26°26'41"N, 127°54'39"E), 15 m depth, 23 April 2009 (NSMT–Cr 22249). Paratype: 1 female, ex body cavity of *Trapania miltabrancha*, off Red Beach, Kin, Okinawa-jima Island, North Pacific Ocean, Japan (26°26'41"N, 127°54'39"E), 15 m depth, 29 May 2008 (RUMF–ZC–02105).

##### Type locality.

off Red Beach, Kin, Okinawa-jima Island, North Pacific Ocean, Japan (26°26'41"N, 127°54'39"E).

##### Description of holotype female.

**Figure 8.**
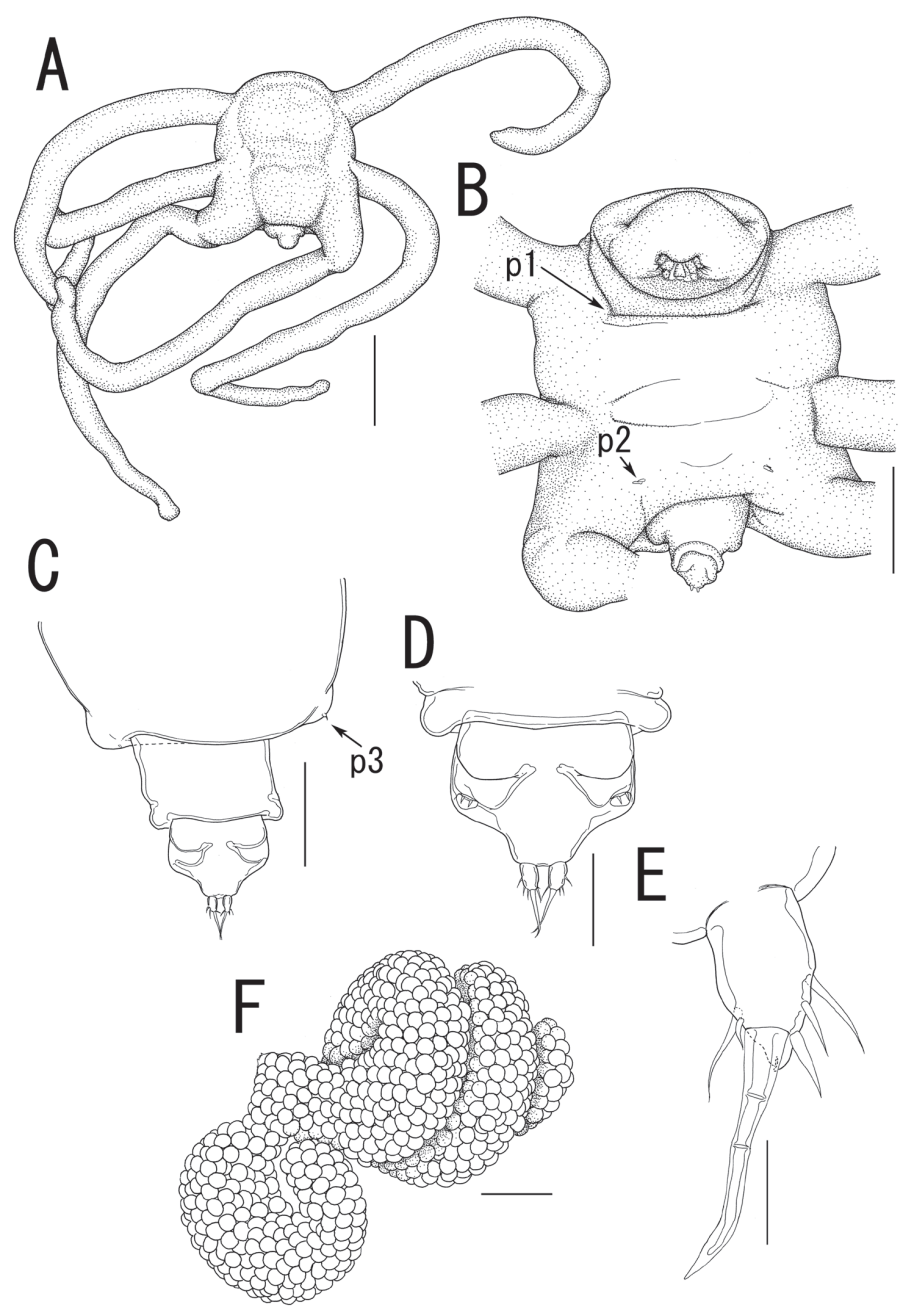
*Splanchnotrophus imagawai* sp. n., female, holotype NSMT–Cr 22249. **A** habitus, dorsal **B** habitus, ventral, p1 = leg 1, p2 = leg 2 **C** posterior portion of body, ventral, p3 = leg 3 **D** fourth pedigerous somite and genito-abdomen, ventral **E** caudal ramus, ventral **F** egg sac. Scale bars = 1 mm in **A**; 500 μm in **B, F**; 200 μm in **C**; 100 μm in **D**; 20 μm in **E**.

**Figure 9.**
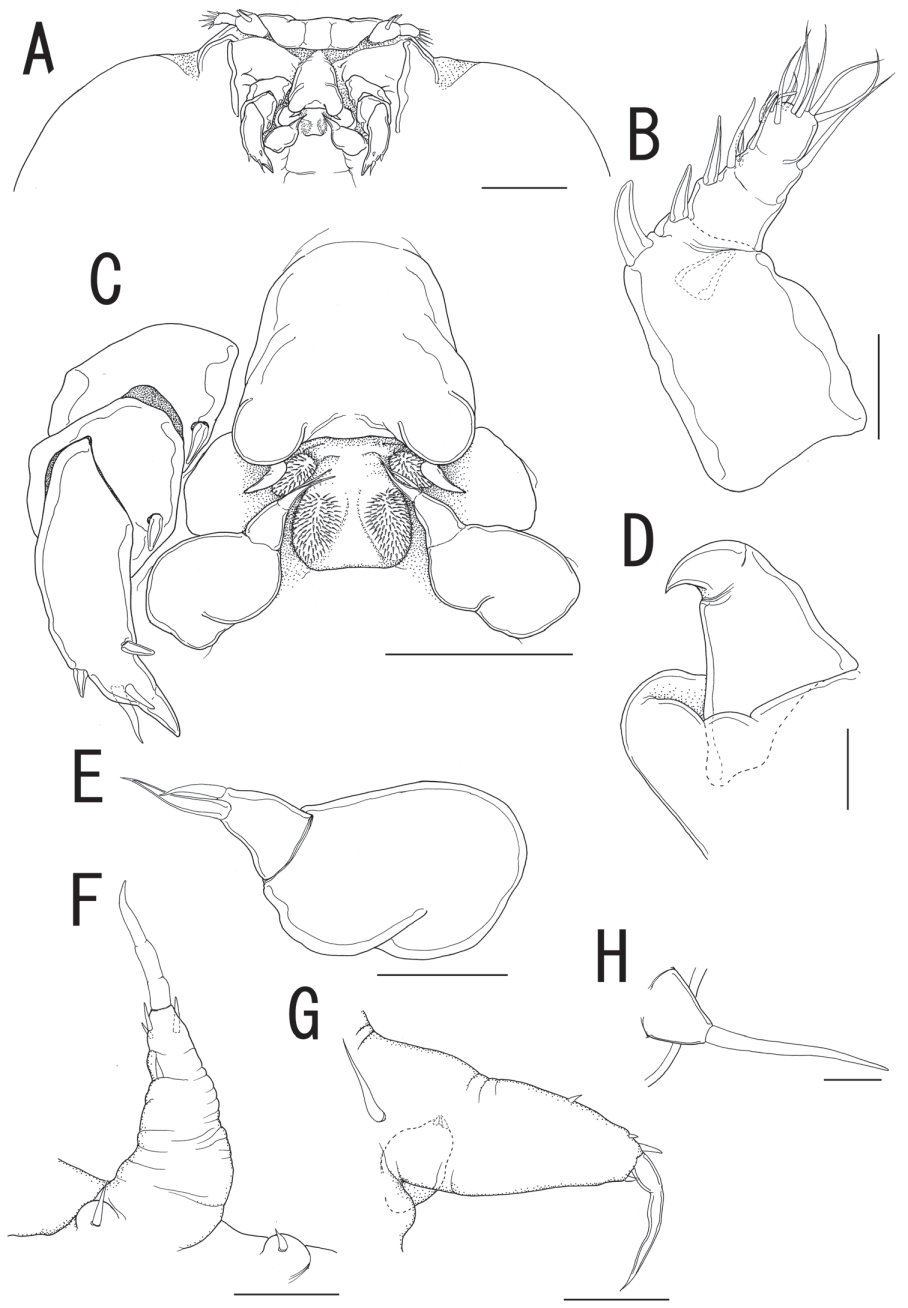
*Splanchnotrophus imagawai* sp. n., female, holotype NSMT–Cr 22249. **A** anterior portion of cephalosome **B** antennule, ventral **C** oral area **D** mandible, posterior **E** maxilla **F** leg 1 **G** leg 2 **H** leg 3. Scale bars = 100 μm in **A**; 50 μm in **B, C**; 10 μm in **D, H**; 20 μm in **E, F, G**.

Body (Figure 8A) 1.86 long, composed of swollen prosome and small 2-segmented urosome. Prosome composed of anterior region as cephalosome, middle region comprising first and second pedigerous somites, and posterior region as third pedigerous somite. Cephalosome (Figure 8B) not elongated, broad and unarmed with protruded rostral region (Figure 9A). Middle region (Figure 8B) compact, about as wide as long, bearing 3 pairs of lateral processes, without posterolateral processes. Posterior region (Figure 8B, C) broad, bearing paired bulbs carrying leg 3 on tip. Lateral processes (Figure 8A) long and slender, about twice as long as body length. Urosome (Figure 8C, D) small; genito-abdomen ampulla-like posterior portion bearing paired apertures without posterolateral lobes; opercula bearing small shield-like structure with 2 spiniform processes. Caudal ramus (Figure 8E) small, about 1.5 times as long as wide, bearing 6 setae and 2 dorsal spiniform processes; apical seta long, styliform.

Antennule (Figure 9A, B) 2-segmented; terminal segment divided by 2 constrictions making it appearing as original segmentation; proximal segment bearing 2 blunt spines; terminal segment bearing 2 blunt spines and 1 seta in proximal part, 3 setae and 1 blunt element in middle part, and 9 setae and 2 blunt elements in distal part. Antenna (Figure 9A, C) 3-segmented; coxo-basis broad, bearing 1 medial spine; proximal segment of endopod bearing 1 medial spine; terminal segment of endopod drawn out into strong apical claw, with 2 spines and 2 setal elements. Labrum (Figure 9A, C) bilobate, bearing flat surface. Mandible (Figure 9A, C, D) spatulate, tapering into single curved blade without dentiform processes. Labium (Figure 9A, C) with two patches of spinules. Maxillule not observed. Paragnath (Figure 9C) represented by pinnate lobe. Maxilla (Figure 9C, E) 2-segmented; syncoxa unarmed; allobasis tapering into spiniform process and bearing seta. Maxilliped absent.

Legs 1 and 2 (Figures 8B, 9F, G) unsegmented, weakly sclerotized; protopod bearing outer basal seta, largely incorporated into ventral wall of prosome; elongate exopodal lobe separated from small endopodal lobe; exopodal lobe drawn out into long process bearing multiple constrictions, wrinkly surface, and 4 setal elements; endopodal lobe bulbous, bearing spiniform apical element. Leg 3 (Figures 8C, 9H) represented by conical process with apical seta.

Egg sacs (Figure 8F) bilobate, bearing curved side and spiral side; dull white in live color (Figure 1E, F).

*Variation of female morphology*. The morphology of the female paratype is as in the holotype. The specimens from type series (n = 2) range from 0.71-1.86 (1.28 ± 0.81) BL.

##### Male.

Unknown.

##### Site.

Female specimens were found in the body cavity of host nudibranchs. They grasped the host’s visceral sac by the lateral processes. Only the posterior tip of the urosome and the egg sacs were exposed from the host’s gill circle (Figure 1E, F).

##### Etymology.

The specific name “*imagawai*” honours the collector of this new species, Mr. Kaoru Imagawa who is a professional diver. The discovery of the new species was brought by his extraordinary ability to find small nudibranch gastropods.

##### Remarks.

The female of the new species differs from *Splanchnotrophus angulatus*, *Splanchnotrophus dellachiajei*, *Splanchnotrophus gacilis* and *Splanchnotrophus willemi* in the absence of posterolateral processes on the prosome (vs. present, see Hancock and Norman 1863; Canu 1891; Delamare Deboutteville 1950; Huys 2001). *Splanchnotrophus helianthus* sp. n. lacks such processes but differs clearly from the new species in the following characters in females: the anterior region of the prosome is elongate and bent to ventral (vs. not elongate); the middle region of the prosome has a constriction posterior to the base of the first lateral processes (vs. without constriction); the genito-abdomen possesses posterolateral lobes (vs. without lobes); the mandible bears dentiform processes (vs. without processes); the endopodal lobe of leg 1 is adpressed to the exopodal lobe via the small protopod (vs. the endopodal lobe separated from the exopodal lobe); and leg 3 is located on the third pedigerous somite directly (vs. leg 3 located on the apex of paired bulbs).

#### Majimun gen. n.

urn:lsid:zoobank.org:act:844ACF3B-5676-4961-A013-4731BE2023FB

http://species-id.net/wiki/Majimun

##### Diagnosis of adult female.

Body elongate, comprising long prosome with 3 pairs of lateral processes and 3-segmented urosome. Prosome composed of anterior region (cephalosome), middle region (including first and second pedigerous somites), and posterior region (including third and fourth pedigerous somites). Cephalosome elongate. Middle region elongate, about twice as long as wide, without posterolateral processes. Posterior region elongate. Lateral processes long and slender. Urosome small; genital double somite cylindrical, narrower at mid region, bearing paired apertures with slightly prominent posterolateral corners; opercula bearing small shield-like structure with 2 spiniform processes. Caudal rami small, bearing 6 setae; apical seta styliform.

Antennule 3-segmented bearing spiniform elements; proximal segment subdivided into basal part with 4 spines and distal part with 3 elements; middle segment bearing 3 elements; terminal segment bearing 11 elements. Antenna 3-segmented; coxo-basis broad, bearing 1 medial spiniform element; proximal segment of endopod bearing 1 inner spiniform element; terminal segment of endopod drawn out into strong apical claw, with 4 and 1 elements along outer and inner margins, respectively. Labrum bilobate, bearing paired extra lobes and small central, conical protrusion. Mandible spatulate, drawn out into blade with 3 dentiform processes. Maxillule not observed. Paragnath bulbous lobe. Maxilla 2-segmented; syncoxa unarmed; allobasis tapering into curved process, with seta. Maxilliped absent. Labium bearing single pointed process, small paired protrusions ornamented with spinules, and posterolateral patches of spinules.

Legs 1 and 2 composed of protopod largely incorporated into ventral wall of prosome, with exopodal and endopodal lobes; protopod bearing outer basal seta, small protrusion at base of endopodal lobe of leg 1; exopodal lobe indistinctly 2-segmented, tapering into spiniform apical process; endopodal lobe cylindrical bearing apical process. Leg 3 represented by conical process with apical seta.

Egg sacs cylindrical and spiral.

##### Diagnosis of adult male.

Body cyclopiform, composed of cephalothorax and 5 cylindrical somites. Cephalothorax large, bulbous, incorporating first and second pedigerous somites, bearing transverse constriction posterior to mouth parts and paired posterolateral outgrowth. Genital somite bearing paired apertures; opercula unarmed. Caudal rami conical, about as long as wide, bearing 6 setae; apical seta styliform. No marked sexual dimorphism in antennule and mouth parts. Shape of antenna as in female except terminal endopodal segment bearing 5 elements; inner margin bearing 2 of 5 elements. Mandible elongate, drawn out into spatulate apical blade with 3 dentiform processes.

Legs 1 and 2 composed of round protopod with outer basal seta, indistinctly 2-segmented exopodal lobe drawn out into pointed process, and non-segmented endopodal segment with apical small process. Leg 3 represented by conical process with apical seta.

##### Type and only species.

*Majimun shirakawai* sp. n. by the present designation.

##### Etymology.

The generic name, “*majimun*”, refers to a dialect in Okinawa, which means demons. The gender is neuter.

##### Remarks.

Females of *Lomanoticola* and *Splanchnotrophus* differ from *Majimun* gen. n. in having a 2-segmented urosome comprising the genital double somite and the anal somite (vs. a 3-segmented urosome and the genital double somite separated from the abdomen) (Huys 2001; present study). Females of *Ismaila* spp. share a 3-segmented urosome, which includes 1 postgenital somite (vs. 2 somites). There are also differences in the following female characters between *Ismaila* and the new genus: the antennule is 2-segmented (vs. 3-segmented); the mandible consists of a small rod tipped with a short tooth and a slender spine (vs. drawn out into a blade with dentiform processes); the paragnath is absent (vs. present); the maxillule is made of a lobe with 2 setae (vs. absent); and the maxilla has the allobasis drawn out into a multipinnate endite with 2 accessory elements (vs. with the allobasis drawn out into a curved process with 1 seta)(see Ho 1981; Haumayr and Schrödl 2003). *Majimun* gen. n. does not show distinct sexual dimorphism in the antennule, the antenna, and the mouth parts. The new genus also possesses a 3-segmented antennule, the mandible, and the paragnath in both sexes and 2 postgenital somites in females. These characters are not shared with *Arthurius* (see Huys 2001). All of these characters are shared with *Ceratosomicola*, except the 3-segmented antennule. However, *Ceratosomicola* differs from the new genus by having the following characters in both sexes: the antennule is composed of 4 distinct segments (vs. 3 segments); the antenna is conical (vs. elongate); the mandible is covered with numerous spinules (vs. spatulate bearing a blade with dentiform processes around apex); and the maxilla possesses a lanceolate process without armature (vs. with process and 1 seta) (see Huys 2001; present study).

#### Majimun shirakawai gen. et sp. n.

New Japanese name: banana-umiushi-yadori for both the genus and the species

urn:lsid:zoobank.org:act:4C7B7C83-FFB4-4997-9329-5999D8553200

http://species-id.net/wiki/Majimun_shirakawai

Figures 1G, H 10 [Fig F11] 12

##### Type material.

Holotype: female, ex body cavity of *Roboastra luteolineata* (Baba) (Nudibranchia: Polyceridae), off Miyagi Beach, Chatan, Okinawa-jima Island, East China Sea, Japan (26°19'44"N, 127°44'35"E), 6 m depth, 14 June 2010 (NSMT–Cr 22250). Allotype: male (NSMT–Cr 22251) collection data same as that of holotype. Paratypes: 1 female and 1 male, ex body cavity of *Roboastra gracilis* (Bergh), off Cape Maeda, Onna, Okinawa-jima Island, East China Sea, Japan (26°26'41"N, 127°46'20"E), 5 m depth, June 2010 (NSMT–Cr 22252); 2 females and 1 male, ex body cavity of *Roboastra luteolineata* (Baba), off Miyagi Beach, Chatan, Okinawa-jima Island, East China Sea, Japan (26°19'44"N, 127°44'35"E), unknown water depth, 14 October 2009 (RUMF–ZC–02106).

##### Type locality.

off Miyagi Beach, Chatan, Okinawa-jima Island, East China Sea, Japan (26°19'44"N, 127°44'35"E).

##### Description of adult female.

**Figure 10.**
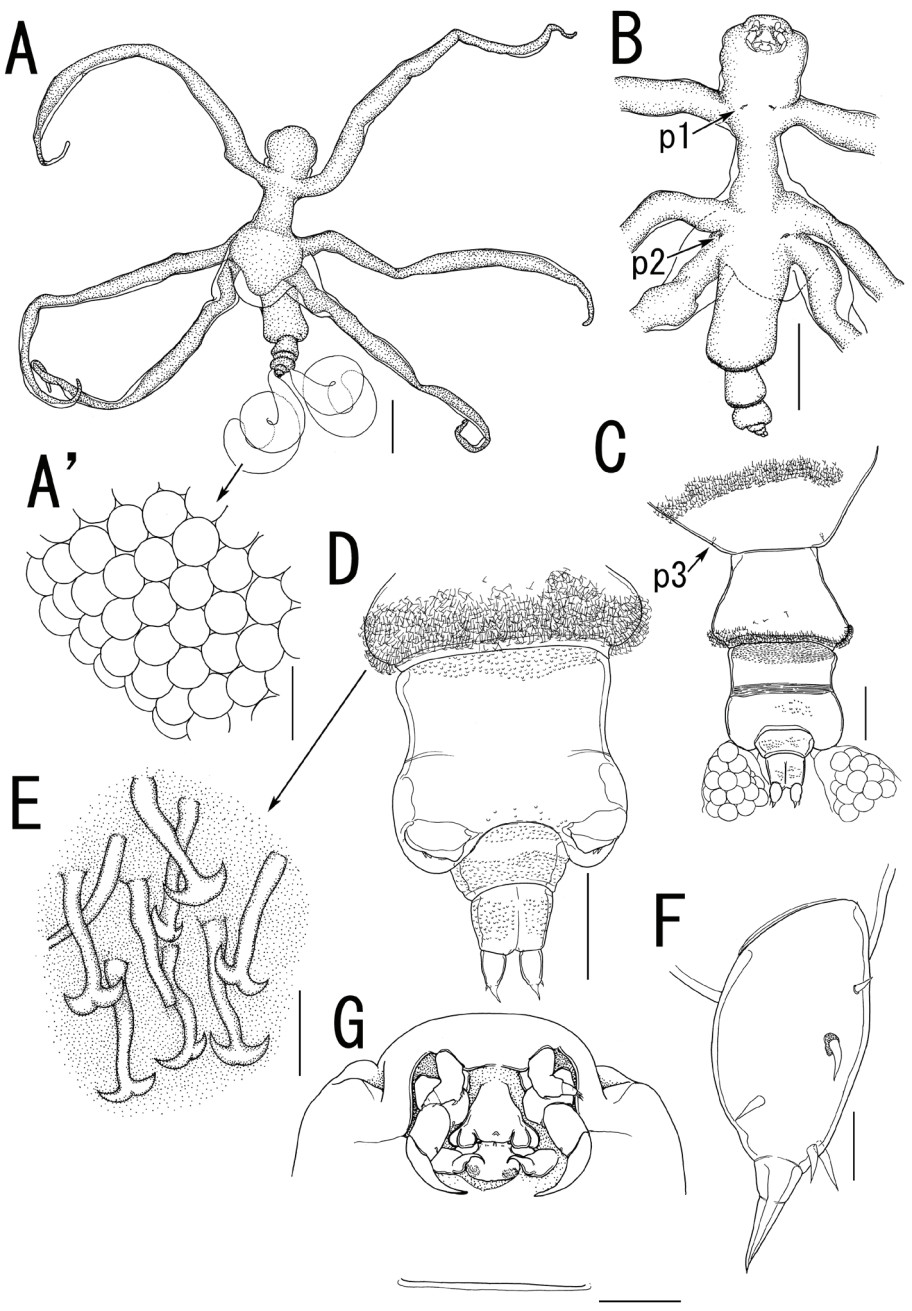
*Majimun shirakawai* gen. et sp. n., female, holotype NSMT–Cr 22250. **A** habitus, dorsal **A’ **enlarged view of egg sac **B** habitus, ventral, p1 = leg 1, p2 = leg 2 **C** posterior portion of body, dorsal, p3 = leg 3 **D** posterior portion of fourth pedigerous somite and genito-abdomen, ventral **E** enlarged view of the patch of anchor-like spinules on posterior margin of fourth pedigerous somite **F** caudal ramus, ventral **F** anterior portion of cephalosome, ventral. Scale bars = 1 mm in **A, B**; 100 μm in **A’**; 200 μm in **C, D, G**; 20 μm in **E, F**.

Body length (Figure 10A) 4.99, elongate, composed of elongate prosome with 3 pairs of lateral processes and 3-segmented urosome. Prosome composed of anterior region (cephalosome), middle region (comprising first and second pedigerous somites), and posterior region (comprising third and fourth pedigerous somites). Cephalosome rectangular (Figure 10A, B), bearing protruded rostral area (Figure 10G). Middle region (Figure 10A, B) elongate, about twice as long as wide, bearing constriction at base of first lateral processes and dorsal posterior lobe, without posterolateral processes. Posterior region (Figure 10A, B) elongate, third and fourth pedigerous somites covered with anchor-shaped spinules (Figure 10E) along posterior margin (Figure 10C, D). Lateral processes (Figure 10A) long and slender, about 1.3 times as long as body length. Urosome (Figure 10C, D) small; genital double somite cylindrical, narrower at middle length, bearing paired apertures with slightly prominent posterolateral corners; opercula bearing small shield-like structure with 2 spiniform processes. Caudal ramus (Figure 10F) small, fusiform, about twice as long as wide, bearing 6 setae; apical seta styliform.

**Figure 11. F11:**
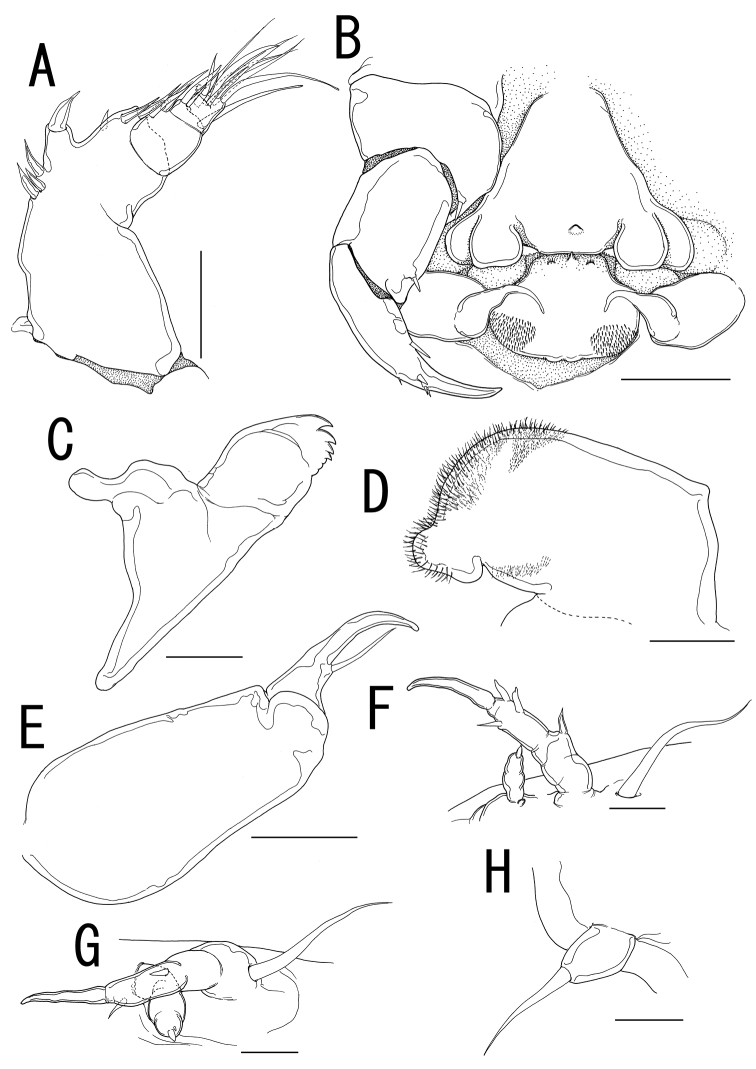
*Majimun shirakawai* gen. et sp. n., female, holotype NSMT–Cr 22250. **A** antennule, anterior **B** oral area **C** mandible **D** paragnath **E** maxilla **F** leg 1 **G** leg 2 **H** leg 3. Scale bars = 50 μm in **A, E**; 100 μm in **B**; 20 μm in **C, D, F, G**; 10 μm in **H**.

Antennule (Figure 11A) 3-segmented bearing spiniform elements; proximal segment subdivided basal part with 4 elements and distal part with 3 elements; middle segment bearing 3 elements; terminal segment bearing 11 elements. Antenna (Figure 11B) 3-segmented; coxo-basis broad, bearing 1 inner spiniform element; proximal segment of endopod bearing 1 inner spiniform element; terminal segment of endopod drawn out into strong apical claw, with 4 and 1 elements along outer and inner margins, respectively. Labrum (Figure 11B) bilobate, bearing paired extra lobes along posterior margin and small central, conical protrusion. Mandible (Figure 11B, C) spatulate, drawn out into blade with pointed tip and 3 dentiform processes. Maxillule not observed. Paragnath (Figure 11B, D) bulbous lobe covered with setules. Maxilla (Figure 11B, E) 2-segmented; syncoxa unarmed; allobasis tapering into curved process, with seta. Maxilliped absent. Labium (Figure 11B) bearing single pointed process, small paired protrusions ornamented with spinules, and posterolateral patch of spinules.

Legs 1 and 2 (Figures 10B, 11F, G) composed of protopod largely incorporated into ventral wall of prosome with exopodal and endopodal lobes; protopod bearing outer basal seta, small protrusion at base of endopodal lobe of leg 1; exopodal lobe indistinctly 2-segmented, tapering into spiniform apical process, bearing 4 and 2 elements in legs 1 and 2, respectively; endopodal lobe cylindrical bearing apical process. Leg 3 (Figures 10C, 11H) represented by conical process with apical seta.

Egg sacs (Figure 10A) cylindrical and spiral; orange in live color (Figure 1G, H).

*Variation of female morphology*.The morphology of body parts of female paratypes is as in the holotype. The specimens from type series (n = 4) range from 3.31-4.99 (3.99 ± 0.77) BL.

##### Description of adult male.

**Figure 12.**
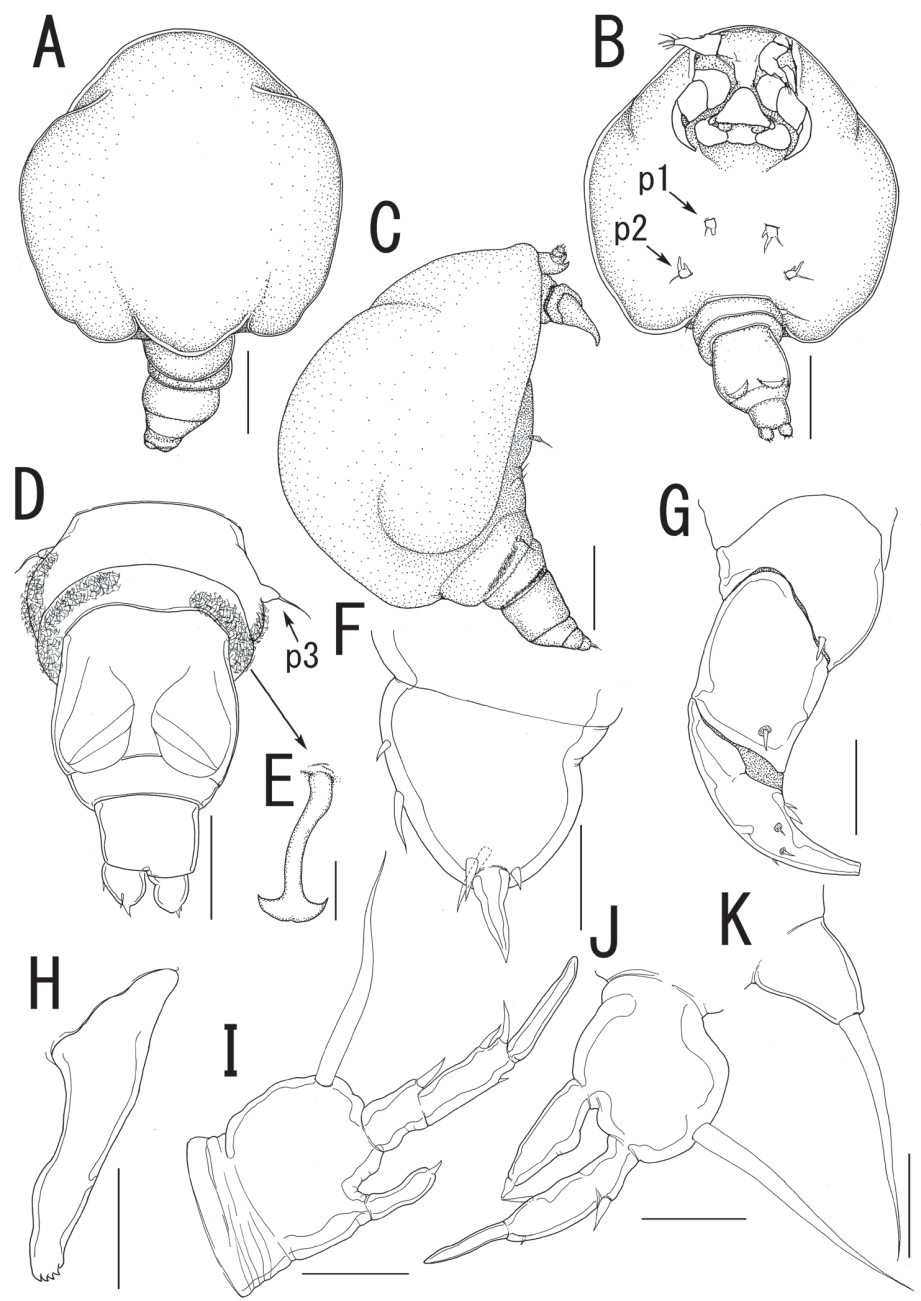
*Majimun shirakawai* gen. et sp. n., male, allotype NSMT–Cr 22251. **A** habitus, dorsal **B** habitus, ventral, p1 = leg 1, p2 = leg 2 **C** habitus lateral **D** posterior portion of body, ventral, p3 = leg 3 **E** anchor-like spinule on posterior margin of fourth pedigerous somite **F** caudal ramus, ventral **G** antenna, anterior **H** mandible **I** leg 1 **J** leg 2 **K** leg 3. Scale bars = 200 μm in **A, B, C**; 100 μm in **D**; 20 μm in **F, H, I, J, K**; 10 μm in **E**; 50 μm in **G**.

Body (Figure 12A–C) 1.02 long, cyclopiform, composed of cephalothorax and 5 cylindrical somites. Cephalothorax (Figure 12A–C) large, bulbous, incorporating first and second pedigerous somites, bearing transverse constriction posterior to mouthparts and paired posterolateral outgrowths. Posterior margin of third and fourth pedigerous somites (Figure 12C, D) covered with anchor-shaped spinules (Figure 12E) on both dorsal and ventral surface. Genital somite (Figure 12D) bearing paired apertures; opercula unarmed. Caudal ramus (Figure 12F) conical, about as long as wide, bearing 6 setae; apical seta styliform. No marked sexual dimorphism in antennule and mouthparts. Shape of antenna (Figure 12G) as in female except terminal endopodal segment bearing 5 elements; inner margin bearing 2 of 5 elements. Mandible (Figure 12H) elongate, drawn out into spatulate apical blade with 3 dentiform processes.

Legs 1 and 2 (Figure 12B, I, J) composed of round protopod with outer basal seta, indistinctly 2-segmented exopodal lobe drawn out into pointed process with 4 and 3 elements on legs 1 and 2, respectively, and non-segmented endopodal segment with apical small process. Leg 3 (Figure 12D, K) represented by conical process with apical seta.

*Variation of male morphology*. The morphology of male paratypes is as in the allotype. The specimens from type series (n = 3) range from 0.50-1.02 (0.75 ± 0.26) in BL.

##### Site.

All specimens of both sexes were found in the body cavity of the host nudibranchs. The lateral processes on the prosome of females grasped the host’s visceral sac, and their mouthparts were in touch with the host’s gonads. The posterior tip of the urosome and the egg sacs were exposed from the posterior region of the host’s gill circle (Figure 1G). Males were attached to the posterior part of the female prosome (Figure 1H). Both females and males bear patches of hook-like spinules (Figures 10E, 12E) on the posterior margin of the third and fourth pedigerous somites.

##### Etymology.

The new species is named after Mr. Naoki Shirakawa, an expert diver who finds remarkable animals. He collected the nudibranchs infected by the new species.

## Discussion

Despite the fact that Splanchnotrophidae comprises 23 species in 5 genera, only 4 species in 3 genera have been recorded from the North Pacific Ocean: 2 species of *Ismaila* from the East coast and another 2 species in 2 other genera from the western North Pacific (Huys 2001; Salmen et al. 2008a). One species of *Ceratosomicola* has been reported from Japanese waters (Fujita 1895; see Huys 2001) and this species is described herein as *Ceratosomicola japonica* sp. n. The other species, *Arthurius bunakenensis* Salmen, Kaligis, Mamangkey & Schrödl, 2008 was described from Gangga Island off northern Sulawesi, Indonesia (Salmen et al. 2008a). With the descriptions of 4 new species and 1 new genus in this paper, there are now at least 4 species in 3 genera of splanchnotrophids in Japanese waters and 5 species in 4 genera in the western North Pacific Ocean.

Currently, 4 species of *Splanchnotrophus* are recognized, and all of them have been described or reported from European waters (Huys 2001). Thus, *Splanchnotrophus helianthus* sp. n. and *Splanchnotrophus imagawai* sp. n. are the first and second species found from North Pacific Ocean. On the other hand, the host nudibranchs of these 2 new species, *Thecacera pennigera* and *Trapania miltabrancha*, are widely distributed: *Trapania pennigera* is known from the subtropical Atlantic and Pacific region (Debelius and Kuiter 1998), and *Thecacera miltabrancha* is probably widely distributed in the Indo-West Pacific because this species was originally described from Indonesia (Gosliner and Fahey 2008). We infer that the 2 *Splanchnotrophus* spp. described in this paper are not distributed only in a limited region. In fact, with the recent spread of SCUBA diving, many opisthobranch gastropods infected by splanchnotrophids have been found and their pictures have been taken from temperate to tropical waters around the world. Because of the shape of the egg sac, some of such splanchnotrophids are surmised to be undescribed species of *Splanchnotrophus*.

The original descriptions of the 4 known species of *Splanchnotrophus* lack adequate illustrations of mouthparts and the swimming legs (Hancock and Norman 1863; Canu 1891; Hecht 1893; Delamare Deboutteville 1950). Therefore, the shape of the prosome and the urosome has been used for species identification, and the shape of the genito-abdomen is especially important. Nevertheless, some characters in the mouthparts and the swimming legs are useful to separate species of *Splanchnotrophus* from each other: the mandibles of *Splanchnotrophus angulatus*, *Splanchnotrophus gracilis*, and *Splanchnotrophus helianthus* sp. n. carry several dentiform processes on the apical parts (Huys 2001; present study) but that of *Splanchnotrophus imagawai* sp. n. lack such armature. The shape of legs 1 and 2 in females also differs between *Splanchnotrophus angulatus*, *Splanchnotrophus helianthus* sp. n., and *Splanchnotrophus imagawai* sp. n. *Splanchnotrophus* spp. have been described based on female specimens, and male specimens were described only for 2 species, *Splanchnotrophus angulatus* and *Splanchnotrophus gracilis* (Hancock and Norman 1863; Huys 2001). *Splanchnotrophus helianthus* sp. n. is a third species described based on both sexes, and the male anal somite of this new species nearly completely withdrawn into genital somite is not shared with the other 2 species. It is, therefore, considered that the male morphology can also serve as useful character to distinguish between morphologically similar species of *Splanchnotrophus*.

*Majimun* gen. n. and *Ceratosomicola* share 2-segmented postgenital somites, cylindrical egg sacs, and posterolateral lobes on the male’s prosome (see Huys 2001; present study). On the other hand, *Majimun* gen. n. shares the following characters in both sexes with *Splanchnotrophus*: the antenna has elongate middle and terminal segments; the mandible bears a blade that is not recurved and not covered with spinules; the paragnath is present; the maxilla is 2-segmented with a seta; legs 1 and 2 possess exopodal and endopodal lobes; and leg 3 is represented by a conical projection with 1 apical seta (see Huys 2001; present study). *Ceratosomicola* has a globular caudal ramus with 5 short elements, but that of *Splanchnotrophus* is elongate with 5 setae and 1 terminal spiniform seta. The somewhat elongate, fusiform caudal ramus with 5 minute and 1 short spiniform terminal setae of *Majimun* gen. n. shows just an intermediate type between that of *Ceratosomicola* and *Splanchnotrophus*. However, the antennule of *Majimun* gen. n. is 3-segmented, which differs from that of both genera. Huys (2001) mentioned that the ancestral splanchnotrophid antennule is 5-segmented, and the proximal segment in *Ceratosomicola* is homologous to the first 2 segments in ancestral one. In *Majimun* gen. n., the proximal segment is large and bears 4 spines and 3 elements on the proximal and distal parts, respectively, and this segment also corresponds to the first 3 segments in the ancestral antennule.

### Key to genera of Splanchnotrophidae, based on females:

**Table:** 

1	Postgenital somites at most 1-segmented	2
–	Postgenital somites 2-segmented	5
2	Prosome with 1 pair of relatively small anteroventral processes; antennule short, 1-segmented; mandible absent	*Arthurius*
–	Prosome without paired anteroventral processes; antennule at least 2-segmented; mandible present	3
3	Prosome with 1 elongate dorsal process; antennule 2-segmented; mandible represented by single rod with 2 elements on tip	*Ismaila*
–	Prosome without such dorsal processes; antennule indistinctly 4-segmented; mandible drawn out into curved blade with or without dentiform processes	4
4	Egg sacs attached at midlength with well-developed anterior and posterior lobes; prosome with lateral processes longer than body	*Splanchnotrophus*
–	Egg sacs attached at subterminally, cylindrical, and slightly curved; prosome with conical lateral processes shorter than or as long as body	*Lomanoticola*
5	Antennule 3-segmented; antenna with elongate middle segments; mandible drawn out into spatulate apical blade with dentiform processes	*Majimun* gen. n.
–	Antennule 4-segmented; antenna conical with short middle segments; mandible represented by incurved blade covered with numerous spinules	*Ceratosomicola*

## Supplementary Material

XML Treatment for Ceratosomicola japonica

XML Treatment for Splanchnotrophus helianthus

XML Treatment for Splanchnotrophus imagawai

XML Treatment for Majimun

XML Treatment for Majimun shirakawai
